# ‘How Poor Are You?’ – A Comparison of Four Questionnaire Delivery Modes for Assessing Socio-Economic Position in Rural Zimbabwe

**DOI:** 10.1371/journal.pone.0074977

**Published:** 2013-09-20

**Authors:** Sophie J. S. Pascoe, James R. Hargreaves, Lisa F. Langhaug, Richard J. Hayes, Frances M. Cowan

**Affiliations:** 1 London School of Hygiene and Tropical Medicine, London, United Kingdom; 2 Royal Free and University College Medical School, London, United Kingdom; 3 Department of Community Medicine, University of Zimbabwe, Harare, Zimbabwe; Tulane University School of Public Health and Tropical Medicine, United States of America

## Abstract

**Background:**

Assessing socio-economic position can be difficult, particularly in developing countries. Collection of socio-economic data usually relies on interviewer-administered questionnaires, but there is little research exploring how questionnaire delivery mode (QDM) influences reporting of these indicators. This paper reports on results of a trial of four QDMs, and the effect of mode on poverty reporting.

**Methods:**

This trial was nested within a community-randomised trial of an adolescent reproductive health intervention conducted in rural Zimbabwe. Participants were randomly allocated to one of four QDMs (three different self-administered modes and one interviewer-administered mode); a subset was randomly selected to complete the questionnaire twice. Questions covered three socio-economic domains: i) ownership of sellable and fixed assets; ii) ability to afford essential items; and iii) food sufficiency. Statistical analyses assessed the association between QDM and reporting of poverty, and compared the extent of response agreement between questionnaire rounds.

**Results:**

96% (n = 1483) of those eligible took part; 395 completed the questionnaire twice. Reported levels of poverty were high. Respondents using self-administered modes were more likely to report being unable to afford essential items and having insufficient food. Among those completing the questionnaire twice using different modes, higher levels of poverty and food insufficiency were reported when they completed the questionnaire using a self-administered mode.

**Conclusion:**

These data suggest that QDM plays a significant role in how different socio-economic indicators are reported, and reminds us to consider the mode of collection when identifying indicators to determine socio-economic position.

## Introduction

Health inequalities in low and middle-income countries are a public health problem of growing importance. Monitoring trends in health inequalities and comparing their magnitude between settings should help identify strategies to reduce them and requires accurate data on economic status [1]–[4] If the methods used to ascertain economic status do not accurately measure the poverty or wealth status of those living within a community, then this may distort observed relationships, possibly leading to incorrect policy recommendations.

Assessing socio-economic position (SEP) within developing countries can be difficult. The term ‘poverty’ covers a wide range of concepts and constructs and can vary across context and time. Income and/or consumption expenditure data are generally considered gold standard quantitative measures of SEP; there are however, disadvantages and difficulties with using these indicators. Data can be complex and time-consuming to collect and subject to potential reporting biases [2], [5] Many studies have instead assessed SEP using an asset-based approach, combining data on ownership of fixed and sellable assets such as type of toilet, plough, bicycle as a single measure of SEP. [6]–[9] Others have included data on food sufficiency or ability to afford essential items (such as health care and education). [6], [8], [10]–[13].

Data to inform an asset-based measure of SEP are almost always collected using interviewer-administered questionnaires. [6], [8], [14], [15] This allows interviewers to clarify questions, probe for more information and provide reassurance around sensitive or stigmatizing questions. Reporting level of wealth is sensitive, and may be affected by social desirability bias. Recent comparative research on the effect of questionnaire delivery mode (QDM) on collection of data on sensitive variables, such as sexual behaviour and mental health, indicate that self-administered methods (which includes paper and computer self-administered questionnaires) generally lead to higher, and possibly more accurate, rates of reporting. [16]–[20] Tourangeau et al. found reporting of income and SEP to be an equally sensitive experience, yet in our search of the literature we found no comparative research exploring the influence of survey method on reporting of SEP within developing countries. [16].

The Regai Dzive Shiri project was a community-randomized trial of a multi-component adolescent reproductive health intervention conducted in rural Zimbabwe between 2003–2007. [21] In 2006 we nested a randomised controlled trial of four QDM into the interim survey in order to compare reporting of various stigmatized behaviours and responses to sensitive questions by mode. We report here on differences in prevalence of indicators of SEP between the different modes.

## Methods

### Ethical Approval

This study received approval from the Medical Research Council of Zimbabwe and the ethics committees at University College London and the London School of Hygiene and Tropical Medicine.

**Figure 1:**
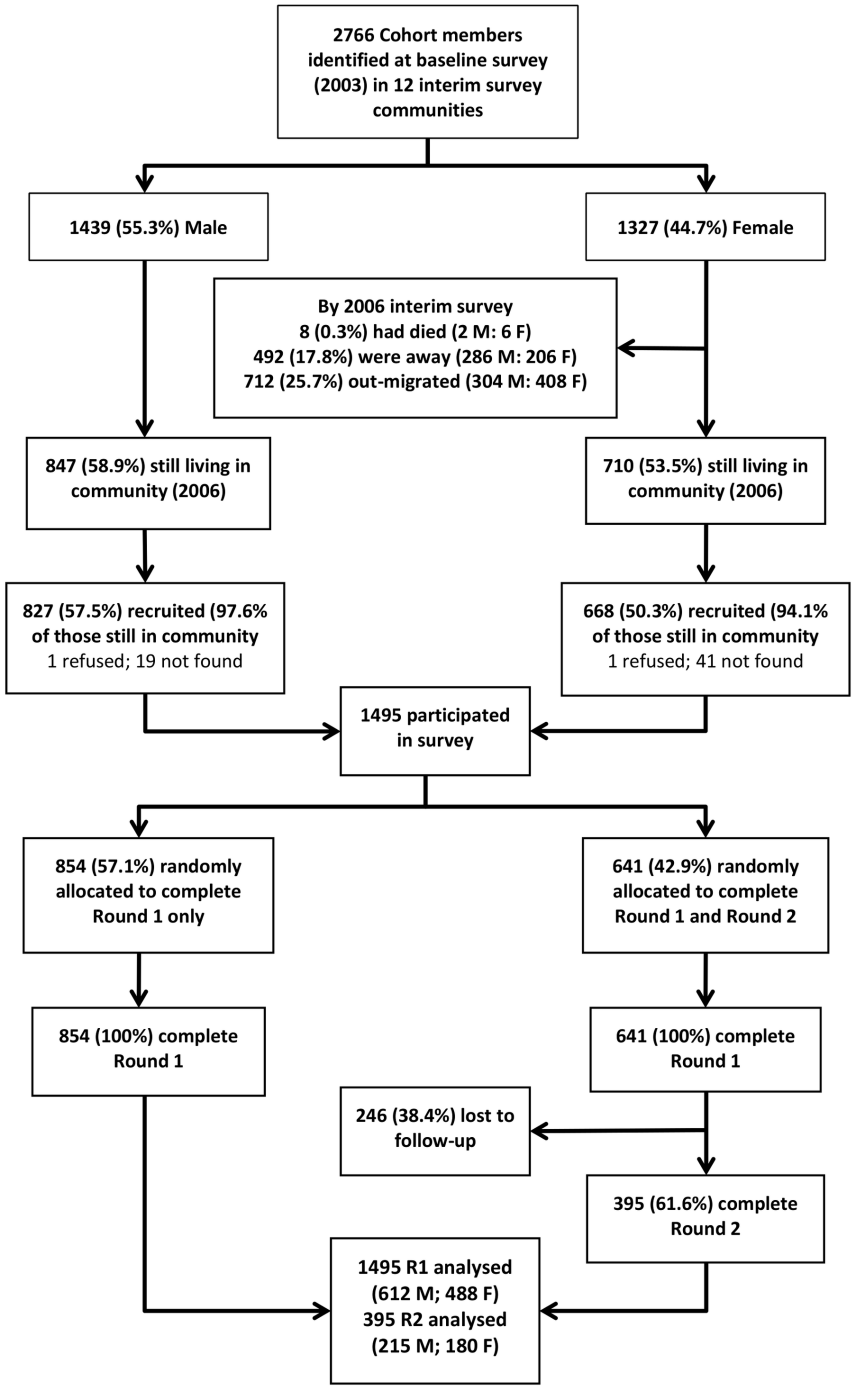
Flowchart to describe participant recruitment and allocation to questionnaire delivery methods.

In 2003, the Regai Dzive Shiri baseline survey was conducted in 30 rural communities in three provinces in eastern Zimbabwe. Eighty-six percent (n = 6791) of pupils in their ninth year of schooling (mean age 15yrs) attending 82 secondary schools in the trial communities took part. [21] In 2006, we conducted a further survey in 12 of the 30 communities that were randomly selected. Survey respondents were eligible if they were members of the trial cohort and were still resident in these communities. Using a permuted random block design, survey respondents were randomly allocated to one of four QDM: i) interviewer-administered questionnaire (IAQ), ii) self-administered questionnaire (SAQ) using paper and pen, iii) audio self-administered questionnaire (AudioSAQ) - SAQ accompanied by an audio soundtrack, and; iv) audio computer-assisted self-interview (ACASI) - completed on a laptop computer accompanied by an audio soundtrack (Figure 1).

In order to allow for some internal comparison, two rounds of the questionnaire were administered to just under 30% randomly selected survey respondents. Round 2 was a shortened version of the first questionnaire. Respondents assigned to complete the questionnaire twice were randomly allocated a QDM for Round 2 and asked to return to the survey site one week later to complete Round 2.

The questionnaire covered a wide range of domains including demographics, poverty, mobility, reproductive health knowledge and sexual behaviour. SEP was examined through a series of questions prefaced by ‘Think about yourself and your household now’ and covering three socio-economic domains: i) ownership of sellable assets (e.g. oxcart, blankets) and fixed assets which related to physical household structure (i.e. roof and building materials); ii) ability to afford essential items (e.g. cooking oil, soap, clinic fees); and iii) food sufficiency in the past week (e.g. days without food or skipping meals). These data referred to characteristics of the respondent’s household. Questions were translated into Shona (indigenous language) and cognitive interviewing (a technique used to pre-test question wording) was used to ensure comprehension. [22], [23].

### Data Handling and Analysis

All questionnaire data completed on IAQ, SAQ, and AudioSAQ were double-entered onto a password-protected database. Data from ACASI were downloaded and imported directly into the database.

Chi-square tests were used and risk ratios calculated to compare reporting of each socio-economic indicator between the four QDMs (taking IAQ as the reference group).

A summary variable was then created for each socio-economic domain. For asset ownership and essential items, summed-score indices were created. If a respondent owned an asset or was able to afford an item, this conferred a score of 1; not owning the asset or being able to afford the item was given a score of 0. Indices were created by adding variable scores together in each domain (i.e. the essential item summed-score included 7 variables; asset ownership score 13 variables). These indices were then split into three approximately equal-sized groups (terciles) for the whole population, creating three categories of SEP for each domain (where SEP1 was the poorest category and SEP3 the wealthiest). Chi-square tests and ordinal logistic regression models were used to assess the association between QDM and SEP. One-way analysis of variance (ANOVA) was performed to compare the mean summed-score of each index between QDM. Food sufficiency status was determined based on 4 question items (see tables); food insufficiency was defined by an affirmative response to one or more of these questions. Chi-square tests and logistic regression were used to assess the association between QDM and reported food insufficiency.

For respondents who completed both rounds of the survey, responses were compared between rounds 1 and 2 for all economic indicators included on both questionnaires (this excluded asset-based questions). McNemar’s chi-square test was used to test the null hypothesis of no difference in reporting between rounds. The extent of agreement between rounds was assessed using the kappa statistic. For these analyses, responses were compared between self-administered modes of delivery (i.e. SAQ, AudioSAQ and ACASI) and IAQ; and also compared where respondents completed IAQ in both rounds or a self-administered mode in both rounds. Self-administered modes were combined into one category to restrict the number of comparisons made and allow a more general assessment of differences in reporting between self-administered and interview-administered QDMs. The correlation between essential item scores was explored for those with two rounds of data using different modes in each round. All statistical analyses were performed using Stata 10 (College Station, TX).

## Results

Of 1,557 cohort respondents still living in the study communities at the time of the survey, 96% (n = 1495; 827 males) took part in the interim survey (mean age 18.2yrs; range 15–23); 12 respondents failed to complete the questionnaire. Table 1 presents demographic data; randomization was successful in achieving relative balance of key characteristics across the four arms of the study. The majority of respondents (87%; 1277) had completed at least four years of secondary school (ie completed Form 4); just over 4% (63) were married; and levels of orphaning were high with 40% (573/1432; 95% CI: 37.5–42.6) reporting the loss of one or both parents (11% had lost both parents). [18], [24].

**Table 1:** Demographic characteristics by questionnaire delivery mode.

	Questionnaire delivery mode					
Characteristic	IAQ^1^	SAQ^2^	AudioSAQ^3^		ACASI^4^	Total
	(n = 364)	(n = 368)	(n = 376)	(n = 375)		(n = 1483)
Mean age (years) [Standard deviation]	18.2 [1.15]	18.2 [1.16]	18.2 [1.14]	18.2 [1.12]		18.2 [1.14]
% Male	54.1	56.0	57.4	53.6		55.3
% Never been married	94.7	95.5	95.2	97.3		95.7
% completed Form 4	86.5	81.0	89.3	89.3		86.6
Orphaning						
Maternal orphan	8.9	5.7	8.1	5.0		6.9
Paternal orphan	24.9	19.1	21.0	23.1		22.1
Dual orphan	12.2	7.8	11.5	12.4		11
Proportion living in each district (%)						
Mashonaland - Chikomba	13.5	15.0	17.6	15.5		15.4
Manica - Buhera	21.7	21.2	20.0	20.0		20.7
Manica - Makoni	10.2	11.1	9.6	10.7		10.4
Masvingo - Gutu	25.3	22.3	23.4	23.7		23.7
Masvingo - Zaka	17.3	18.2	17.3	17.6		17.6
Masvingo - Masvingo rural	12.1	12.2	12.2	12.5		12.3

1 IAQ = Interviewer administered questionnaire;

2 SAQ = Self-administered questionnaire;

3 AudioSAQ = Audio self-administered questionnaire;

4 ACASI = Audio-computer assisted self-interview.

### Socio-economic Indicators and Questionnaire Delivery Mode

For the 1483 that completed the questionnaire, completion of socio-economic questions was high with less than 2% item non-response rate. Table 2 presents the proportion of respondents reporting each item in each socio-economic domain by QDM. Overall levels of reported poverty were high. Most households appeared to own everyday items such as tables, chairs and sufficient blankets, although 10% of participants reported that their household did not own these items. Only 10% of participants reported owning luxury items such as a motorcycle, car, or fridge, and less than half owned an oxcart or bicycle. Households of over 55% of participant were unable to afford cooking oil, 21% unable to afford soap to wash clothes; 41% unable to afford clinic fees; and 55% reported school absenteeism due to lack of money for fees. Reports of food insufficiency were also high with over 18% reporting going a day without food in the past week as there was no food in the household, and 28% unable to afford more than one meal a day. Overall, 45% of respondents reported one or more indicators of food insufficiency.

**Table 2:** Proportion of respondents reporting each socio-economic indicator and risk ratio by questionnaire delivery mode.

		% reporting each indicator					Risk Ratio [95% CI]			
Indicator	n	IAQ^1^	SAQ^2^	AudioSAQ^3^	ACASI^4^	Chi-squarep-value	IAQ^1^	SAQ^2^	AudioSAQ^3^	ACASI^4^
		(n = 364)	(n = 368)	(n = 376)	(n = 375)		(n = 364)	(n = 368)	(n = 376)	(n = 375)
**Ownership of sellable and fixed assets:**										
*Sellable assets - does not own:*										
Oxcart	1483	61.3	61.4	56.1	54.4	p = 0.12	1.00	1.00 [0.83–1.20]	0.92 [0.89–1.12]	0.89 [0.78–1.00]
Bicycle	1483	56.0	61.1	57.7	54.7	p = 0.32	1.00	1.09 [0.97–1.17]	1.03 [0.91–1.17]	0.98 [0.86–1.11]
Motorcycle or car	1483	91.8	88.7	89.1	88.5	p = 0.46	1.00	0.97 [0.92–1.01]	0.97 [0.93–1.02]	0.96 [0.92–1.01]
Plough	1483	17.3	18.5	13.0	16.0	p = 0.21	1.00	1.07 [0.78–1.46]	0.75–0.53–1.06]	0.92 [0.67–1.28]
Radio	1483	36.5	39.1	38.8	41.1	p = 0.66	1.00	1.07 [0.89–1.29]	1.06 [0.88–1.28]	1.12 [0.94–1.35]
Fridge	1483	93.7	89.4	88.8	90.4	p = 0.12	1.00	0.95 [0.91–1.00]	0.95 [0.91–0.99]	0.96 [0.92–1.01]
TV	1483	70.9	75.3	72.1	71.2	p = 0.53	1.00	1.06 [0.97–1.16]	1.02 [0.93–1.11]	1.00 [0.92–1.10]
Phone	1483	90.1	83.7	85.4	84.3	p = 0.05	1.00	0.93 [0.88–0.98]	0.95 [0.90–1.00]	0.94 [0.88–0.99]
Any everyday items (blankets/tables/chairs)	1483	3.8	14.4	9.0	12.3	p<0.001	1.00	3.74 [2.12–6.63]	2.35 [1.28–4.31]	3.19 [1.78–5.70]
*Fixed assets (physical structure of household):*										
Bush used for toilet (vs. blair/flush toilet)	1476	28.2	32.3	29.4	32.5	p = 0.49	1.00	1.15 [0.92–1.43]	1.04 [0.83–1.31]	1.15 [0.93–1.44]
House built with poles and dagga (vs. bricks/cement)	1481	0.8	4.4	2.9	3.5	p = 0.03	1.00	5.28 [1.55–17.95]	3.57 [1.00–12.69]	4.21 [1.21–14.64]
Grass roof (vs. tin/asbestos/tiles)	1476	24.2	35.7	30.3	36.3	p = 0.001	1.00	1.48 [1.18–1.86]	1.25 [0.99–1.59]	1.50 [1.20–1.88]
Dirt floors (vs. cement)	1480	10.4	16.9	17.9	16.0	p = 0.02	1.00	1.62 [1.11–2.37]	1.71 [1.18–2.48]	1.53 [1.05–2.24]
**Ability to afford essential items:**										
Cannot afford:										
to cook with oil at each meal	1483	45.0	62.5	56.4	56.3	p<0.001	1.00	1.39 [1.21–1.59]	1.25 [1.08–1.44]	1.25 [1.08–1.44]
to eat meat or fish at least 4x/wk	1483	67.9	75.0	71.3	71.5	p = 0.21	1.00	1.11 [1.01–1.21]	1.05 [0.95–1.16]	1.05 [0.96–1.16]
to drink tea once/day	1483	40.4	45.1	44.7	45.6	p = 0.46	1.00	1.12 [0.94–1.32]	1.11 [0.94–1.31]	1.13 [0.96–1.33]
soap to wash clothes	1483	13.5	25.8	21.0	20.0	p = 0.001	1.00	1.92 [1.40–2.62]	1.56 [1.13–2.16]	1.49 [1.07–2.07]
to pay clinic fees if sick	1483	31.8	50.0	40.4	38.9	p<0.001	1.00	1.57 [1.31–1.88]	1.27 [1.04–1.54]	1.22 [1.00–1.49]
All in household do not own at least 1 pair of shoes	1483	27.8	45.6	37.5	42.4	p<0.001	1.00	1.65 [1.35–2.01]	1.35 [1.09–1.67]	1.53 [1.25–1.87]
Have been absent from school because no money for fees	1483	56.3	56.0	54.3	55.7	p = 0.94	1.00	0.99 [0.87–1.13]	0.96 [0.84–1.10]	0.99 [0.87–1.12]
**Food sufficiency:**										
Adult skipped a meal in last week	1466	10.2	26.1	22.6	22.1	p<0.001	1.00	2.56 [1.80–3.64]	2.21 [1.54–3.17]	2.17 [1.52–3.11]
Gone a day without food in the last week	1473	10.4	23.0	21.2	19.7	p<0.001	1.00	2.20 [1.54–3.14]	2.03 [1.42–2.90]	1.89 [1.31–2.72]
Sometimes I go to bed hungry	1483	14.3	22.0	17.0	18.4	p = 0.05	1.00	1.54 [1.12–2.11]	1.19 [0.85–1.67]	1.29 [0.93–1.79]
Cannot afford to eat more than one meal a day	1483	23.4	32.1	29.0	27.2	p = 0.07	1.00	1.37 [1.09–1.74]	1.24 [0.97–1.58]	1.16 [0.91–1.49]

1 IAQ = Interviewer administered questionnaire;

2 SAQ = Self-administered questionnaire;

3 AudioSAQ = Audio self-administered questionnaire;

4 ACASI = Audio-computer assisted self-interview.

The data show relatively little difference in the proportion of households reporting ownership of sellable assets between QDMs, but substantial differences for the other domains. Higher rates of reporting an inability to afford essential items and food insufficiency were seen among participants who used self-administered modes of questionnaire delivery.

The right half of Table 2 presents risk ratios to compare the proportion reporting each indicator between the four QDMs. Those using self-administered modes were between two and four times as likely to report not owning blankets, tables and chairs, and were more likely to report that their house was built of traditional materials (poles and dagga and grass roof), than those using IAQ. Likewise, in the domain ‘ability to afford essential items’, respondents using self-administered modes were more likely to report being unable to afford certain items than IAQ users. For example, SAQ users were 1.9 times more likely to report not being able to afford clothes soap as IAQ users; AudioSAQ and ACASI users were 1.6 and 1.5 times more likely to report this item respectively. There was also a strong association between QDM and reporting food insufficiency; respondents using self-administered modes were twice as likely to report adults skipping meals and going a day without food as respondents who were interviewed.

### Association between Socio-economic Position and Questionnaire Delivery Mode

Table 3 shows the proportion of respondents categorised in each SEP tercile for each socio-economic domain by QDM; the mean summed-score for both the asset wealth and essential item index by delivery mode; and odds ratios to demonstrate the relationship between SEP and QDM. Where SEP was defined by ownership of fixed and sellable assets, whilst there was some evidence of a difference in reported asset wealth between the different QDMs (p = 0.016); the mean index score was similar irrespective of which mode was used (p = 0.218); and odds ratios indicated that the odds of being categorised in the wealthier SEP categories versus the poorest category were similar for all QDMs.

**Table 3:** Relationship between classification of socio-economic position and questionnaire delivery mode for each socio-economic domain.

	% in each SEP category				p-value	OR [95% CI]^7^						
Socio-economic position (SEP)	IAQ^1^	SAQ^2^	AudioSAQ^3^	ACASI^4^		IAQ^1^	SAQ^2^		AudioSAQ^3^		ACASI^4^	
*Asset wealth score (n):*	*360*	*360*	*371*	*375*		1.00	0.73 [0.56–0.95]		0.94 [0.72–1.23]		0.84 [0.64–1.09]	
SEP 1 (Poorest - score 0.0–0.46)	35.0	43.6	38.8	43.7								
SEP 2 (Score 0.47–0.62)	35.8	32.8	30.7	25.6								
SEP 3 (Score 0.63–1.0)	29.2	23.6	30.5	30.7	p = 0.016 5							
Mean score [SD]	0.55 [0.17]	0.52 [0.21]	0.55 [0.20]	0.54 [0.21]	p = 0.218 6							
*Ability to afford essential items (n):*	*364*	*368*	*376*	*375*		1.00	0.53 [0.40–0.69]		0.68 [0.52–0.88]		0.68 [0.52–0.89]	
SEP 1 (Poorest - score 0.0–0.43)	33.5	50.3	43.4	45.3								
SEP 2 (Score 0.44–0.71]	36.0	29.9	33.5	28.8								
SEP 3 (Score 0.72–1.00)	30.5	19.8	23.1	25.9	p<0.001 5							
Mean score [SD]	0.60 [0.28]	0.49 [0.30]	0.53 [0.29]	0.53 [0.30]	p<0.001 6							
*Food sufficiency (n):*	*363*	*359*	*368*	*375*								
Insufficient food	35.0	50.1	48.1	46.9	p<0.001 5	1.00	1.87 [1.39–2.52]		1.72 [1.28–2.32]		1.64 [1.22–2.21]	

1 IAQ = Interviewer administered questionnaire;

2 SAQ = Self-administered questionnaire;

3 AudioSAQ = Audio self-administered questionnaire;

4 ACASI = Audio-computer assisted self-interview.

5 Chi-square test;

6 Oneway analysis of variance *F-test*;

7 Crude odds ratio calculated using ordinal logistic regression for asset wealth and essential item SEP scores.

The latter section of the table though, presents strong evidence of an association between QDM and SEP defined by ability to afford essential items, and with reporting food sufficiency. The data showed that those using self-administered modes were more likely to be categorised in the poorest SEP tercile, and to report having insufficient food, than IAQ users. ANOVA showed a significant difference in the mean essential item score between the QDMs (p<0.001) with higher mean sum-scores among IAQ users. Odds ratios indicated that for participants using SAQ to report ability to afford essential items, the odds of being categorised in SEP3 or SEP2 rather than SEP1, were 0.5 times the odds of being categorised in these wealthier categories when using IAQ. Those using AudioSAQ and ACASI were 0.7 times as likely to be categorised in the higher SEP terciles as those using IAQ. The odds of reporting food insufficiency among SAQ users were 1.9 times those of participants who used IAQ; AudioSAQ and ACASI users were also significantly more likely to report food insufficiency (OR = 1.7 and 1.6 respectively).

### Comparison between Round 1 and Round 2

Of the 395 respondents who completed the questionnaire twice, 131 completed the two rounds using IAQ in one round and a self-administered mode in the other (SAQ (n = 45), AudioSAQ (n = 46), ACASI (n = 40)); 30 completed IAQ in both rounds; and 234 completed a self-administered mode in both rounds (27 completed SAQ-SAQ; 23 AudioSAQ-AudioSAQ; 31 ACASI-ACASI; 50 SAQ-AudioSAQ or vice versa; 57 SAQ-ACASI or vice versa; and 46 ACASI-AudioSAQ or vice versa).

Tables 4 and 5 show the proportion that reported each socio-economic indicator by each survey round. Table 4 presents data for respondents who used a different mode in each round i.e. IAQ in round 1 and self-administered in Round 2 or vice versa; Table 5 presents data for respondents who used IAQ or a self-administered mode in both rounds. A greater proportion of respondents reported not being able to afford essential items or aspects of food insecurity when they completed the questionnaire using a self-administered mode, irrespective of whether the self-administered mode was completed in the first or second round. For example, 11% of respondents who used IAQ in Round 1 reported having gone a day without food in the last week (Table 4). However, when these respondents completed the questionnaire using a self-administered mode in Round 2, 22% reported a day without food. Among respondents who first completed the questionnaire using a self-administered mode, 22% reported a day without food; yet only 6% of these respondents reported the same indicator when the questionnaire was interviewer-administered in Round 2. The Kappa statistic (κ) for several indicators (particularly food sufficiency indicators) in Table 4 are less than or equal to 0.4, which suggests that there was generally only poor to moderate agreement in the answers given between the two rounds and two modes of questionnaire.

**Table 4:** Proportion of respondents reporting each socio-economic indicator in each round and the extent of agreement between each round for respondents using IAQ in one round and self-administered in the other.

	(n = 65)		p-value	kappa	(n = 66)		p-value	kappa
Indicator	IAQ R1 a	SA R2 b			SA R1 c	IAQ R2 d		
*Ability to afford essential items:*								
Cannot afford:								
to cook with oil at each meal	41.5	55.4	0.05	0.36	59.1	45.4	0.04	0.55
to eat meat or fish at least 4x/wk	58.5	73.8	0.01	0.53	75.8	74.2	0.80	0.31
to drink tea once/day	40.0	50.8	0.16	0.23	47.0	13.6	<0.001	0.18
soap to wash clothes	12.3	16.9	0.51	0.44	19.7	4.6	0.02	−0.08
to pay clinic fees if sick	35.4	46.2	0.09	0.59	47.0	36.7	0.09	0.48
All in household do not own at least 1 pair of shoes	23.1	44.7	<0.001	0.41	37.9	22.7	0.01	0.51
Been absent from school - no money for fees	49.2	53.8	0.49	0.42	50.0	39.4	0.12	0.54
*Food security:*								
Adult skipped a meal in last week	12.3	24.6	0.02	0.50	27.4	12.9	0.04	0.27
Gone a day without food in the last week	10.8	21.5	0.06	0.39	22.2	6.4	0.01	0.26
Sometimes I go to bed hungry	18.5	27.7	0.18	0.40	21.2	12.1	0.15	0.36
Cannot afford to eat more than one meal/day	21.5	40.0	0.004	0.44	30.3	15.2	0.03	0.25

a Interview-administered questionnaire completed in round 1.

b Self-administered questionnaire mode (i.e. SAQ, AudioSAQ or ACASI) completed in round 2.

c Self-administered questionnaire mode completed in round 1.

d Interview-administered questionnaire mode completed in round 2.

**Table 5:** Proportion of respondents reporting each socio-economic indicator in each round and the extent of agreement between each round for respondents using the same mode of questionnaire delivery in Rounds 1 and 2.

	(n = 30)		p-value	kappa	(n = 234)		p-value	kappa
Indicator	IAQ R1 a	IAQ R2 b			SA R1 c	SA R2 d		
*Ability to afford essential items:*								
Cannot afford:								
to cook with oil at each meal	43.3	50.0	0.62	0.73	59.4	57.7	0.60	0.49
to eat meat or fish at least 4x/wk	70.0	66.7	>0.99	0.46	76.9	75.6	0.64	0.52
to drink tea once/day	50.0	36.7	0.22	0.60	41.9	49.6	0.05	0.30
soap to wash clothes	23.3	16.7	0.62	0.59	27.4	26.1	0.68	0.40
to pay clinic fees if sick	23.3	43.3	0.07	0.42	46.2	43.2	0.28	0.63
All in household do not own at least 1 pair of shoes	26.7	30.0	>0.99	0.75	42.7	44.0	0.65	0.63
Been absent from school - no money for fees	66.7	53.3	0.22	0.59	50.0	47.9	0.57	0.34
*Food security:*								
Adult skipped a meal in last week	13.3	13.3	>0.99	1.00	24.9	22.6	0.41	0.54
Gone a day without food in the last week	13.3	16.7	0.50	0.63	21.1	22.4	0.63	0.48
Sometimes I go to bed hungry	23.3	26.7	>0.99	0.73	21.8	23.1	0.63	0.52
Cannot afford to eat more than one meal/day	23.3	16.7	0.69	0.38	28.6	35.5	0.04	0.39

a Interview-administered questionnaire completed in round 1.

b Interview-administered questionnaire mode completed in round 2.

c Self-administered questionnaire mode (i.e. SAQ, AudioSAQ or ACASI) completed in round 1.

d Self-administered questionnaire mode completed in round 2.

Among respondents who completed either IAQ or a self-administered mode in both rounds, there was, for most socio-economic indicators, no statistically significant difference (p>0.05) in the proportion reporting each indicator in each round (Table 5), indicating that there was more consistency between data across the two rounds when the same or similar QDMs were used in each round.

**Figure 2:**
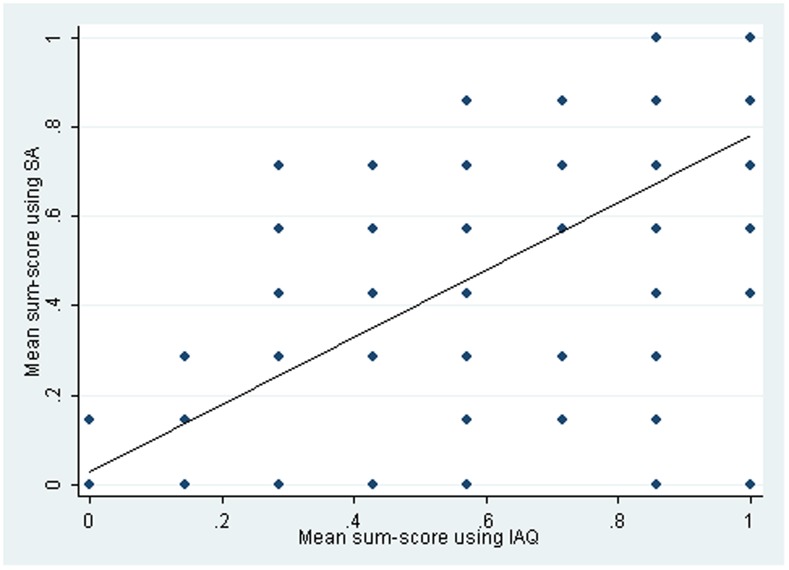
Association between essential items score for those completing two rounds using different modes each round (n = 131).

Figure 2 shows the association between IAQ mean essential item score and self-administered mode score for those who completed two rounds of data using the different modes in each round (n = 131). This demonstrates an association between the scores (r = 0.77; 95% CI: 0.61–0.92; p<0.001), indicating that whilst relative rankings of poverty are preserved with each method, lower scores were obtained using self-administered modes as participants were more likely to report being unable to afford essential items.

## Discussion

We compared the effect of questionnaire delivery mode on reporting of socio-economic indicators in Zimbabwe. Data presented here suggest that QDM is associated with how different socio-economic indicators are reported. While non-item response rate was low for all methods, we found significant differences in prevalence of indicators of economic status between interviewer-administered and self-administered modes. The IAQ was most often associated with the lowest prevalence of reporting items thought to indicate lower SEP, although QDM did not appear to influence reporting of asset wealth to the same degree as reporting ability to afford essential items and food sufficiency. This is reassuring given that many researchers restrict the measurement of SEP to the collection of asset-based indicators, but it does highlight the importance of considering the mode of collection when other indicators are to be measured and recorded. Data comparing Rounds 1 and 2 also showed that, irrespective of the order in which the questionnaires were completed, respondents were more likely to report a higher degree of poverty using self-administered modes, than when questioned by an interviewer. Although there was some inconsistency in reporting of poverty indicators between rounds there was no significant difference in reporting when round alone was taken into account.

This study had a number of strengths, in particular a high coverage within study communities; a large sample size; randomisation of QDM, which enabled us to ensure unbiased comparisons; and repeated rounds, which allowed us to look at the consistency of reporting. However, the context in which this study was undertaken could influence generalisability of the results to other populations. Between 2001 and 2009 Zimbabwe experienced extreme political and economic challenges; hyperinflation and high rates of unemployment and internal displacement may all have impacted on reporting socio-economic status. The restricted age group of the study population, the very high literacy rate in Zimbabwe and the fact that all participants had completed at least some years of secondary education may also have influenced the results, particularly if these younger individuals were less aware of the economic status within their household.

The majority of surveys that examine SEP use a trained interviewer to administer survey tools. One of the main considerations when conducting research on sensitive issues and behaviours is to try and improve validity and reliability of self-reporting by limiting the effects of social desirability bias. [20], [25]–[27] It is generally assumed that higher levels of reporting of socially undesirable attributes, such as lower SEP, indicate more accurate levels of self-reporting. [28]–[31] In this study the prevalence of items indicative of lower SEP appeared to be higher among those using self-administered modes than those being interviewed. However, the expected direction of social desirability bias in this situation is not clear. One possible explanation is that respondents were embarrassed to report their wealth status when asked by an interviewer directly. Qualitative data collected from some of these survey respondents supports this. One female respondent stated: *‘For example, the question ‘Have you ever gone to bed hungry in your household?’ is difficult to answer…because you may just feel embarrassed to say it’s true that in our household we sometimes go to bed hungry.’* [32] Another possibility is that those using self-administered modes of completion felt more able to exaggerate the severity of their economic situation and less censured in their answers. Data from participatory research exploring the issue of wealth, conducted at the same time in these communities indicated that participants censured each other when people tried to claim they were poorer than they actually were (data available from the author). Furthermore, the fact that questionnaire method did not influence reporting of asset wealth could also suggest that people are more honest about items that may be observed or that are objective, but may misreport items that are more subjective or hidden.

These data suggest that there needs to be further exploration of the role of questionnaire delivery mode on self-reported measures of poverty, validated against objective measures where possible, in order to ensure that the measures used to ascertain socio-economic position within a country or community are a true and accurate reflection of actual status.

## References

[1] Barros AJ, Ronsmans C, Axelson H, Loaiza E, Bertoldi AD, et al (2012) Equity in maternal, newborn, and child health interventions in Countdown to 2015: a retrospective review of survey data from 54 countries. Lancet 379: 1225–1233. 22464386 10.1016/S0140-6736(12)60113-5

[2] Howe LD, Galobardes B, Matijasevich A, Gordon D, Johnston D, et al.. (2012) Measuring socio-economic position for epidemiological studies in low- and middle-income countries: a methods of measurement in epidemiology paper. International Journal of Epidemiology. 10.1093/ije/dys037 PMC3396323 22438428

[3] Gwatkin D, Rutstein S, Johnson K, Suliman E, Wagstaff A, et al.. (2007) Socio-Economic Differences in Health, Nutrition and Population in Developing Countries: An Overview.. Washington DC, USA: The World Bank. 18293634

[4] Health CftSDo (2008) Closing the Gap in a Generation: Health Equity through Action on the Social Determinants of Health. Final Report of the Commission on Social Determinants of Health. Geneva: World Health Organization.

[5] Morris SS, Carletto C, Hoddinott J, Christiaensen LJM (2000) Validity of rapid estimates of household wealth and income for health surveys in rural Africa. J Epidemiol Community Health 54: 381–387. 10814660 10.1136/jech.54.5.381 PMC1731675

[6] Central Statistical Office (CSO) [Zimbabwe, Macro International Inc (2007) Zimbabwe Demographic and Health Survey 2005–06. Calverton, Maryland: CSO and Macro International Inc.

[7] Gillespie S, Kadiyala S, Greener R (2007) Is poverty or wealth driving HIV transmission? AIDS 21 Suppl 7S5–S16. 10.1097/01.aids.0000300531.74730.72 18040165

[8] Ministry of Health and Child Welfare (Zimbabwe), Zimbabwe National Family Planning Council, National AIDS Council (Zimbabwe), US Centers for Disease Control and Prevention (2004) The Zimbabwe Young Adult Survey (YAS) 2001–2002 Final Report. Harare, Zimbabwe: Ministry of Health and Child Welfare and U.S. Centres for Disease Control and Prevention.

[9] Wojcicki JM (2005) Socioeconomic status as a risk factor for HIV infection in women in East, Central and Southern Africa: a systematic review. J Biosoc Sci 37: 1–36. 15688569 10.1017/s0021932004006534

[10] Cohen D (1998) Poverty and HIV/AIDS in sub-Saharan Africa. In: SEPED Conference Paper Series - Contribution to a five year review of the Fourth World Conference on Women (Beijing) and the World Summit for Social Development (Copenhagen). 1–12.

[11] Weiser SD, Leiter K, Bangsberg DR, Butler LM, Percy-de Korte F, et al (2007) Food insufficiency is associated with high-risk sexual behavior among women in Botswana and Swaziland.[see comment]. PLoS Medicine 4: 1589–1597. 17958460 10.1371/journal.pmed.0040260 PMC2039764

[12] Rollins N (2007) Food insecurity–a risk factor for HIV infection. PLoS Med 4: 1576–1577. 17958463 10.1371/journal.pmed.0040301 PMC2039757

[13] Central Statistical Office (2000) Zimbabwe Demographic and Health Survey 1999. Central Statistical Office, Harare, Zimbabwe; MEASURE; Macro International Inc.

[14] UNAIDS (2008) 2008 Report on the global AIDS epidemic.

[15] UNDP (2007) Human Development Report 2007/2008. UNDP.

[16] Tourangeau R, Yan T (2007) Sensitive questions in surveys. Psychol Bull 133: 859–883. 17723033 10.1037/0033-2909.133.5.859

[17] Newman JC, Des Jarlais DC, Turner CF, Gribble J, Cooley P, et al (2002) The differential effects of face-to-face and computer interview modes. Am J Public Health 92: 294–297. 11818309 10.2105/ajph.92.2.294 PMC1447060

[18] Langhaug LF, Cheung YB, Pascoe S, Hayes R, Cowan FM (2009) Difference in prevalence of common mental disorder as measured using four questionnaire delivery methods among young people in rural Zimbabwe. J Affect Disord 118: 220–223. 19303145 10.1016/j.jad.2009.02.003 PMC2745501

[19] Gregson S, Zhuwau T, Ndlovu J, Nyamukapa CA (2002) Methods to reduce social desirability bias in sex surveys in low-development settings: experience in Zimbabwe. Sex Transm Dis 29: 568–575. 12370523 10.1097/00007435-200210000-00002

[20] Fenton KA, Johnson AM, McManus S, Erens B (2001) Measuring sexual behaviour: methodological challenges in survey research. Sexually Transmitted Infections 77: 84–92. 11287683 10.1136/sti.77.2.84 PMC1744273

[21] Cowan FM, Pascoe SJ, Langhaug LF, Dirawo J, Chidiya S, et al (2008) The Regai Dzive Shiri Project: a cluster randomised controlled trial to determine the effectiveness of a multi-component community-based HIV prevention intervention for rural youth in Zimbabwe–study design and baseline results. Trop Med Int Health 13: 1235–1244. 18778329 10.1111/j.1365-3156.2008.02137.x

[22] Mavhu W, Langhaug L, Manyonga B, Power R, Cowan F (2008) What is 'sex' exactly? Using cognitive interviewing to improve the validity of sexual behaviour reporting among young people in rural Zimbabwe. Cult Health Sex 10: 563–572. 18649195 10.1080/13691050801948102

[23] Willis GB (2004) Cognitive interviewing: a tool for improving questionnaire design: SAGE. 335 p.

[24] Langhaug LF, Pascoe SJS, Mavhu W, Woelk G, Sherr L, et al (2010) High prevalence of mental ill-health among adolescents living in rural Zimbabwe. Journal of Community Health 35: 355–364. 20571897 10.1007/s10900-010-9261-6 PMC2927984

[25] Catania JA, Gibson DR, Chitwood DD, Coates TJ (1990) Methodological problems in AIDS behavioral research: influences on measurement error and participation bias in studies of sexual behavior. Psychological Bulletin 108: 339–362. 2270232 10.1037/0033-2909.108.3.339

[26] Klimes-Dougan B (1998) Screening for suicidal ideation in children and adolescents: methodological considerations. Journal of Adolescence 21 435–444. 10.1006/jado.1998.0166 9757408

[27] Moum T (1998) Mode of administration and interviewer effects in self-reported symptoms of anxiety and depression. Social Indicators Research 45 279–318.

[28] Brener ND, Billy J, Grady WR (2003) Assessment of factors affecting the validity of self-reported health-risk behaviour among adolescents: evidence from the scientific literature. Journal of Adolescent Health 33 436–457. 10.1016/s1054-139x(03)00052-1 14642706

[29] Kreuter F, Presser S, Tourangeau R (2008) Social desirability bias in CATI, IVR, and web surveys: the effects of mode and question sensitivity. Public Opinion Quarterly 72: 847–865.

[30] Durant LE, Carey MP (2000) Self-administered questionnaires versus face-to-face interviews in assessing sexual behavior in young women. Archives of Sexual Behavior 29: 309–322. 10948721 10.1023/a:1001930202526

[31] Weinhardt LS, Forsyth AD, Carey MP, Jaworski BA, Durant LE (1998) Reliability and validity of self report measures of HIV-related sexual behavior: progress since 1990 and recommendations for research and practice. Archives of Sexual Behavior 27 155–180. 10.1023/a:1018682530519 PMC2452986 9562899

[32] Langhaug LF, Cheung YB, Pascoe SJ, Mavhu W, Chirawu P, et al. (2007) Comparing four questionnaire delivery methods for collection of self-reported sexual behaviour data in rural Zimbabwean youth. In Conference Proceedings of the 17^th^ Biennial meeting of The International Society for Sexually Transmitted Diseases, Seattle, USA. Abstract O−038.

